# A holistic visualization for quality of Chinese materia medica: Structural and metabolic visualization by magnetic resonance imaging

**DOI:** 10.1016/j.jpha.2024.101019

**Published:** 2024-06-08

**Authors:** Jing Wu, Kai Zhong, Hongyi Yang, Peiliang Zhang, Nianjun Yu, Weidong Chen, Na Zhang, Shuangying Gui, Lan Han, Daiyin Peng

**Affiliations:** a College of Pharmacy, Anhui University of Chinese Medicine, Hefei, 230012, China; b Department of Biomedical Engineering, Institute of Advanced Clinical Medicine, Peking University, Beijing, 100191, China; c Hefei Institutes of Physical Science, Chinese Academy of Sciences, Hefei, 230012, China; d Ministry of Education-Anhui Joint Collaborative Innovation Center for Quality Improvement of Anhui Genuine Chinese Medicinal Materials, Anhui University of Chinese Medicine, Hefei, 230012, China

**Keywords:** Chinese materia medica, Quality, Structure and metabolites, Visualization, Magnetic resonance imaging

## Abstract

The quality of Chinese materia medica (CMM) is a challenging and focused topic in the modernization of traditional Chinese medicine (TCM). A profound comprehension of the morphology, structure, active constituents, and dynamic changes during the whole process of CMM growth is essential, which needs highly precise contemporary techniques for in-depth elucidation. Magnetic resonance imaging (MRI) is a cutting-edge tool integrating the benefits of both nuclear magnetic resonance (NMR) spectroscopy and imaging technology. With real-time, non-destructive, and *in situ* detection capabilities, MRI has been previously used for monitoring internal and external structures of plants alongside compounds during physiological processes *in vivo*. Here, factors involved in the holistic quality evaluation of CMMs were investigated. Given the applications of MRI in various plants, several representative CMMs were used as examples to demonstrate a methodology of quality visualization by MRI, embodying holistically monitoring the real-time macroscopic morphology, mesoscopic structure, and microscopic metabolites non-destructively *in situ*. Taken together, the review not only presents a pioneering application mode for utilizing MRI for CMM quality visualization but also holds promise for advancing the quality control and evaluation of CMMs.

## Highlights

• A non-destructive methodology for the constituents-wide and entire-process quality visualization of CMM is established.
• MRI emerges as an optimal technology for real-time monitoring CMM from macro morphology, meso structure, to micro metabolites.
• CMMs of similar origins, species, and processing methods are effectively distinguished by MRI.

## Graphical abstract

**Figure:**
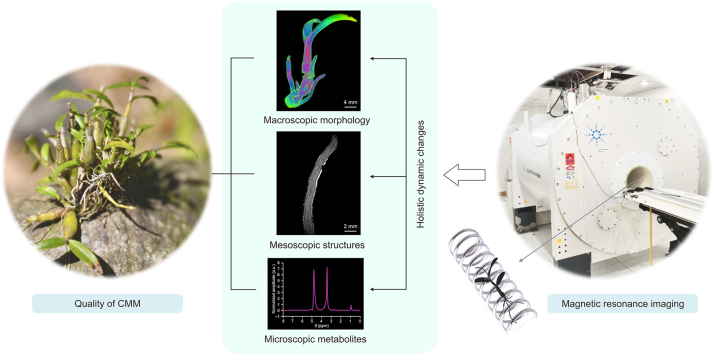

## 1 Introduction

Chinese materia medica (CMM), the indispensable marrow of traditional Chinese medicine (TCM), has occupied a dominant position in combating and mitigating the global challenge of the corona virus disease 2019 (COVID-19). A total of 2711 varieties of CMMs were listed in the Pharmacopoeia of the People's Republic of China (2020 version), representing indispensable constituents of the world's medical arsenal. Each variety harbors a diverse array of potent pharmaceutical compounds [1]. Notably, CMMs exhibit reduced toxicity and fewer adverse effects compared with their synthetic chemical counterparts when employed in disease treatment [2]. Most CMMs come from herbal origins, and many of them are served as daily vegetable supplements on the dining table. Their quality is the primary medical basis for effective prescriptions and clinical efficacy. Genuine CMM quality is intricately influenced by multiple factors, such as germplasm, ecological environment, and harvesting time, eventually reflecting in active metabolites as well as morphological structures [3]. Indeed, disparate environmental conditions precipitate the development of distinct structures and the synthesis of specific bioactive constituents, thereby underpinning the pronounced quality and therapeutic efficacy of CMMs. Consequently, setting up a holistic quality visualization of CMMs will be of great significance for ensuring their clinical efficacy.

Visualization of the quality of CMMs means intuitively showing the various external and internal factors that collectively reflect their species, quality, and efficacy in a holistic manner. These factors encompass, but are not limited to, the morphological structures of the organs and tissues, the distribution of active metabolites, and the overall dynamic changes elicited by temporal and environmental fluctuations, eventually allowing the quality of CMMs to be visualized holistically. Both morphology and spatiotemporal distribution of active metabolites in heterogeneous CMMs can be visualized to provide valuable support for its quality assessment. Conventional detection techniques, reliant solely on simplistic qualitative and quantitative analyses targeting specific active compounds, fall short of meeting the exigencies of TCM's modernization. New strategies integrating multidisciplinary approaches, such as optics and biochemistry, have thus been advanced to address this challenge [4]. In pursuit of the most efficacious means of non-destructive quality visualization of the intricate and multifaceted system of CMMs, access to information in series must rely on the combination of advanced scientific instruments and computer tomography; therefore, it requires the new technology from a holistic point of view to investigate and evaluate the quality of CMMs.

This review raised several aspects influencing the quality of the CMM: external and internal morphology, active metabolites, and their dynamic changes. What we need is to holistically visualize and measure these relevant factors underlying the CMM quality. In the current study, the high-field magnetic resonance imaging (MRI) combined with data from magnetic resonance spectroscopy (MRS) simultaneously is proposed for the first time as a strategy to monitor structures and metabolites in several CMMs. Furthermore, this study endeavors to point out the application potential of MRI in the quality formation and evaluation of CMMs through the holistic visualization and to provide a forward-looking outlook for future development of the pioneering technique.

## 2 Characteristics of MRI and its frontier applications

MRI images are obtained from signals spatially shown by nuclear magnetic resonance (NMR) experiments. MRI possesses a key advantage in performing both static and dynamic measurements. In particular, noteworthy is its capacity to collect data from within samples in a noninvasive way [5]. By this means, the morphology of the internal tissues of any form of an opaque sample can be imaged while allowing a range of chemical parameters to be evaluated. This sets it apart from conventional analytical methods such as liquid chromatography (LC), gas chromatography (GC), and mass spectrometry (MS), which are burdened by complicated pretreatment processes that may precipitate target compound degradation. Several alternative methods for sample visualization exist, including positron emission tomography (PET) [6], confocal laser scanning microscopy [7], optical coherence microscopy [8], optical projection tomography [9], X-rays [10], and mass spectrometry imaging (MSI) [4]. But they cannot study samples in such a holistic and non-destructive way as MRI. The common limitation of all optical techniques is that thick specimens of samples must be processed with organic solvents [11], causing damages to samples and rendering them incompatible with metabolite analysis. MSI, on the other hand, is constrained to surface imaging or tissue sections. On the contrary, MRI transcends such limitations, enabling information acquisition regardless of sample thickness and under non-destructive conditions. A summary of these techniques, alongside their respective strengths and shortcomings, is presented in Table 1.

**Table 1:** A summary of strengths and limitations of techniques mentioned above.

Techniques	Strengths	Limitations
Conventional analysis methods (LC, MS, etc.)	Accurate analysis with good reproducibility and low LODs	Complicated pretreatment, homogenized tissues, and large sample volumes
Near infrared	Simple operation, low cost of detection, and non-destructive samples	Low sensitivity, poor resolution, and minor detection range of compounds
PET	High sensitivity, on-site analysis, and real-time detection	Requiring radiolabeling
MSI	Wide detection range of compounds simultaneously and high resolution (1−100 μm)	Detection of tissue slide, being limited by slicing techniques
MRI	Holistic detection of the whole body *in situ*, non-destructive samples, and fast analysis speed	High cost of equipment and middle resolution (0.05−200 mm)

LC: liquid chromatography; MS: mass spectrometry; LODs: limits of detection; PET: positron emission tomography; MSI: mass spectrometry imaging; MRI: magnetic resonance imaging.

Since the advent of MRI, a large number of applications in plant sciences have come to light. MRI techniques are now available that allow studying root, stem, leaf water content, root anatomy, and (radial and axial) transport in these organs in an integrative way [5,12]. For instance, the quantification of fruit composition in oil palm carried out by Shaarani et al. [13] identified a tissue-specific pattern of oil and water distribution. Similarly, Windt et al. [14] were able to demonstrate that majority of water translocated into the tomato fruit occurs through the xylem rather than the phloem, thus resolving a longstanding challenge in fruit growth modeling. Additionally, MRI has also found applications in the study of certain parameters of fruit quality [15,16].

In summary, MRI offers the capability to image the whole plant and simultaneously monitor water and metabolite dynamics in the plant. This function of MRI is consistent with the holistic research theory of TCM. The overall quality of CMM is reflected in multiple dimensions, which cannot be comprehensively understood by the determination of only one indicator. The challenge lies in locating the intricate distribution and corresponding structures of chemical molecules in the whole plant hinders the acquisition of data for exploring the quality of CMMs. Data generated from MRI can make it possible to investigate the localization of metabolites in heterogeneous tissues of CMMs in a sustainable manner. Attempts made by researchers in this field will put forward the observation of natural compounds in CMMs by MRI. The MRI analysis of multi-constituents CMMs can simplify the time-consuming and complicated pretreatment process for samples. The analysis offers a new strategy for deeper exploration of the quality of CMMs from a holistic perspective. While MRI has been adopted to image structures or measure the composition distribution of certain plants (such as food and crops), only water and some substances related to growth and development, such as sugars, free amino acids, and lipids are currently monitored [17,18]; the distribution of more efficacy-related and quality-responsive compounds in plants has not been reported. Given MRI's capacity to visualize plants from macroscopic to microscopic scales, this study advocates for leveraging MRI to assess the quality of CMMs from a comprehensive perspective encompassing whole body-tissue-metabolite dynamics, thereby achieving holistic visualization of CMM quality.

## 3 Potential and ability of MRI in the holistic visualization of CMM quality

The majority of CMMs are derived from various parts of plants, including roots, stems, leaves, flowers, fruits, and seeds. Therefore, this review examines the applications of MRI in plants and assesses its ability to non-destructively visualize the quality of CMMs, involving monitoring different parts of a single CMM, between similar species, as well as throughout the processing stages. The objective is to demonstrate the feasibility of MRI technology for the comprehensive visualization of CMM quality.

### 3.1 Visualization of the macroscopic morphology and mesoscopic structure

Quality evaluation by morphological identification is a noteworthy method for evaluating the quality of CMMs, as evidenced by the optimal external morphology and internal structure. However, simultaneously acquiring information about both external morphology and internal structure poses a challenge. On one hand, the external morphology is one of the criteria for the quality of CMMs, which is traditionally identified by experience and microscopic identification. Distinguishing between easily confused and similar species becomes difficult. Visualization of morphology can aid in distinguishing similar parts or species and can provide insights into ongoing physiological processes in time for further better accumulating active compounds. On the other hand, imaging internal structures is hard to achieve by optical microscopy due to their opacity. Although thick opaque samples can now be treated with clearing protocols such as solvent-based clearing [11], the depth of optical imaging is still limited to a few hundred microns [19]. Proton (^1^H) NMR images are topological representations of the mobile water binding and portions in soft-tissue samples. The images offer a potentially unique means of gaining access to structural, growth, and hydrodynamic information *in situ* [20]. Since MRI allows three-dimensional (3D) imaging of mesoscopic structures regardless of sample thickness, it compensates for the limitations of optical microscopes in displaying non-destructive internal images of CMMs. In this study, we use MRI to image several typical CMMs, capturing both the overall morphology and the internal structures at the same time.

#### 3.1.1 The whole body

The morphological structure of living plants reflects their growth conditions and is influenced by physiological ecology and the environment. The overall morphology of plants at any given moment determines the physiological processes that are currently taking place, such as photosynthesis [21]. In addition, products of the physiological process embody in the accumulation and distribution of metabolites in CMMs, which are finally the cornerstone of pharmacological functions [22]. It has been reported that the same species of CMM with different morphological forms possessed different active components, leading to different functions in sure [23]. With the aim of improving photosynthetic distribution, the branches and leaf shapes of herbal medicines were pruned and changed to better accumulate active metabolites [24]. Real-time collection of the intact morphological information of CMM and changing it into the optimal morphology to absorb nutrition can benefit healthier growth and better accumulation of active compounds [25].

**Fig. 1:**
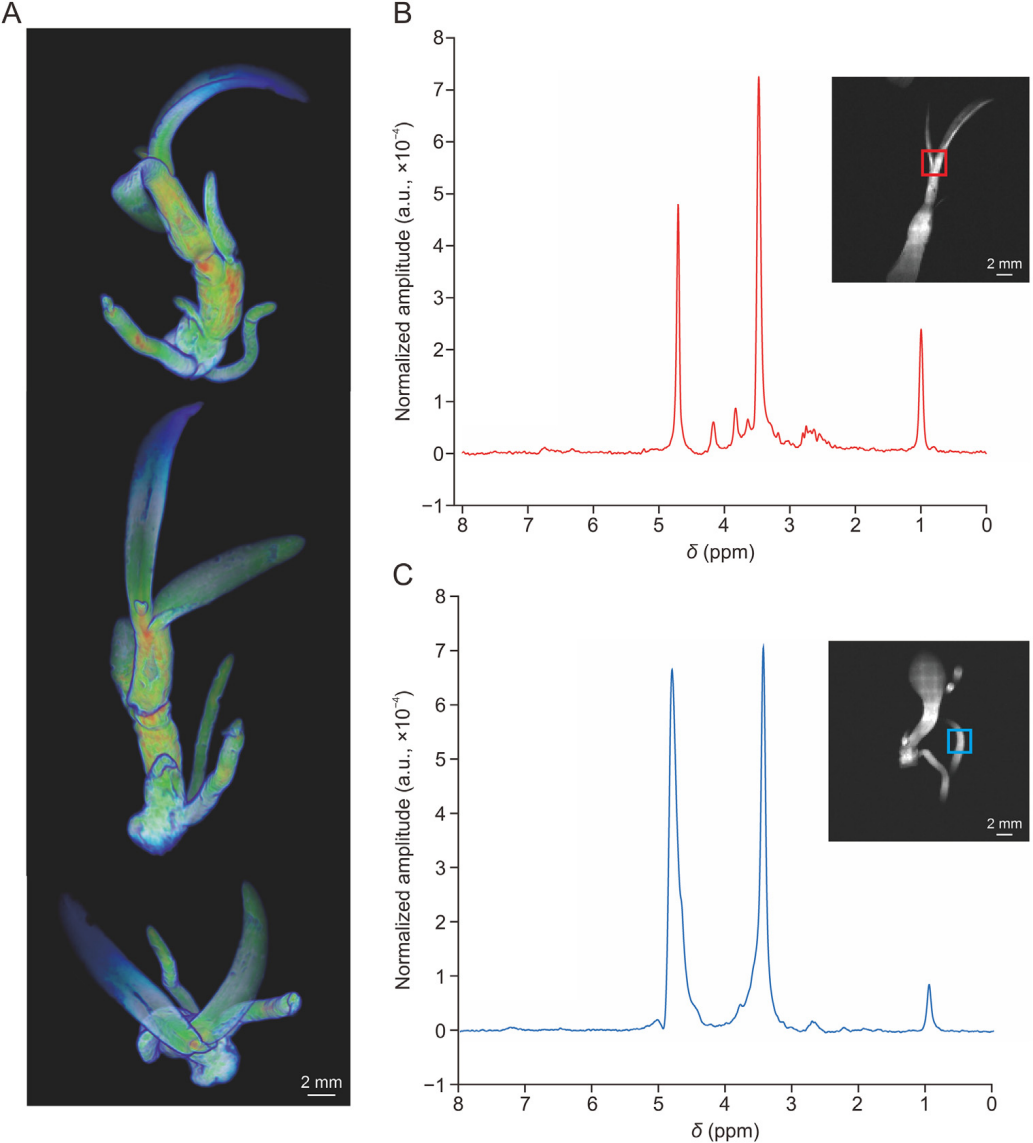
The holistic visualization of *Dendrobium huoshanense* (*D. huoshanense*). (A) The high-resolution three dimensions projection view of the whole plant *D. huoshanense* by magnetic resonance imaging (MRI). (B, C) Metabolic profiling in stem (red) (B) and root (blue) (C) of *D. huoshanense* by magnetic resonance spectroscopy.

The morphology reconstructed by the MRI associated with different growing conditions can promote functional studies of plant quality [26,27]. However, in view of the constraints of small coils and conventional probe heads, visualization for the whole body of CMM is not as easy as for specific organs. Another question worth considering is whether the inherent insensitivity of NMR should be compensated by arranging maximum materials in the coil to obtain an available signal-to-noise ratio (SNR). The improvement of equipment made visualizing the whole plant come true. The whole plant of the small size of hydrophyte *Chamaegigas intrepidus* [28] and the larger tropical liana *Ancistrocladus heyneanus* [29] have been successfully recorded in high-field magnets. The most commonly used approach was constructing an appropriately built coil, as well as a modified probe head for specific species, if needed. In the present study, the microstructure of the whole body and the capsule of the well-known CMM plant *Dendrobium huoshanense* (*D. huoshanense*) were observed with a specific coil and magnet that allowed enough tissues to generate adequate signals (Fig. 1A). In addition to this, information about the compounds contained in specific regions of the imaged object can be obtained simultaneously. The specific regions of root and stem in Fig. 1A were marked out separately as representative displays (Figs. 1B and C). Conventional T_1_-weighted pulse sequence MRI was applied to highlight the free water signal to image internal structures here. In addition to providing conventional T_1_, T_2_, or proton density-weighted structural images, MRI can also provide richer tissue information through more complex imaging methods, such as diffusion-weighted imaging (DTI) [30]. It can provide more compound information based on the signal of water molecules within the tissue, which can be well reflected in the images of tissues through the fitting of physical and mathematical models [31]. The distribution and quantification of several proteins were calculated according to whole-tissue DTI parameters [32]. Some other processing algorithms combined with DTI had been shed light on digging out more measures about the structure and compounds of the detected object [33]. The noninvasiveness of MRI makes it possible to visualize architecture and anatomies of the target CMM that cannot be achieved by conventional microscopy. Such systems have promising potential for providing information on identifying the specific morphology in quite a lot of CMMs in the case of high-resolution NMR imaging studies.

#### 3.1.2 Individual medicinal organs

The organs of CMMs have evolved as medicinal parts, and their quality can be reflected by a range of certain changes in the internal structures during development [17]. In the visualization of the whole body of the CMM morphology mentioned above, the internal structural imaging of individual medicinal organs can also be acquired with MRI simultaneously.

Many of the roots and rhizomes of medicinal plants, where photosynthetic products are stored, can be multifunctional in clinical treatments. The typical Chinese medicine “Gan Cao” is the root and rhizome of the herbal plant *Glycyrrhiza uralensis* [34]. Furthermore, Ginseng Radix et Rhizoma (Ren Shen), Rhizoma Coptidis (Huang Lian), and Salviae Miltiorrhizae Radix et Rhizoma (Dan Shen) are also well-known Chinese medicines for roots and rhizomes [35]. The architecture of roots and their microenvironment can regulate the growth of CMM [36]. Roots absorb nutrients and water to ensure normal development of CMM and multiple active components biosynthesis [37]. Plants can adapt to the external environment, which is partly characterized by changes in root morphology [38]. The spatial distribution, surface area, and architecture of roots may affect the nutrient absorption and compound accumulation of CMM. Accurate clarification of root morphology can help CMM to accumulate active compounds [39]. However, the configuration, sampling, and cleaning of roots may damage their structures, which makes clarifying root morphology difficult [40]. In addition to architecture, growth conditions or diseases inside the root are also hard to observe and deal with [41].

**Fig. 2:**
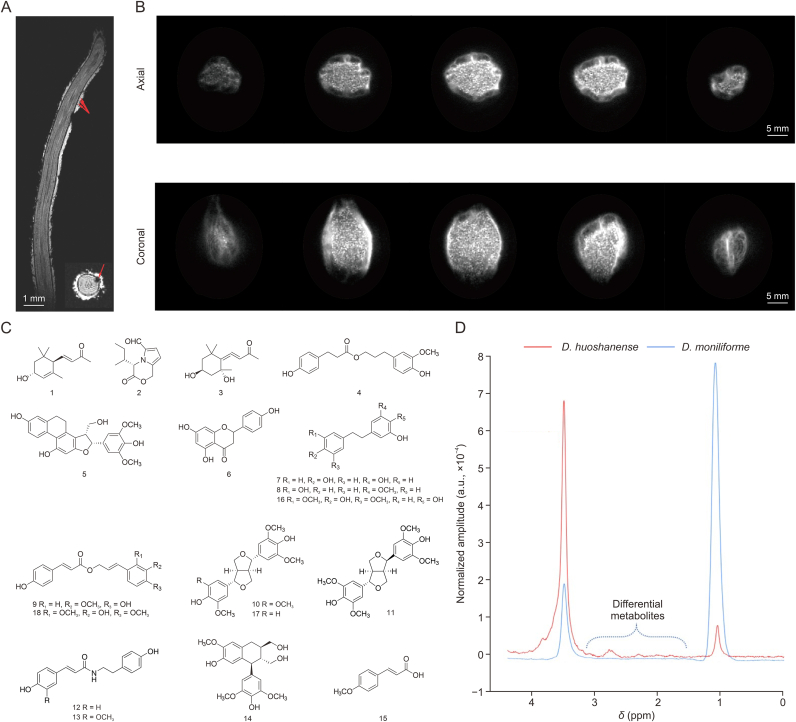
The simultaneous visualization of structure and metabolite profiles of *Dendrobium huoshanense* (*D. huoshanense*). (A) Three dimensions flash magnetic resonance imaging (MRI) with 40-μm isotropic ultra-high resolutions. The arrows point to tiny bubbles inside the roots. (B) Internal images of a very dry capsule of *D. huoshanense* acquired by MRI in axial slices and coronal slices. (C) Chemical structure of differential chemical constituents in stem and root of *D. huoshanense.* (D) Distribution of chemical constituents in roots of *D. huoshanense* and its similar species *Dendrobiummoniliforme* (*D.moniliforme*).

The internal structure reconstruction of various tissues within plants utilizing the MRI can facilitate studies in compound accumulation and distribution. Technological advancements in MRI have made it possible to model the 3D geometry of the rhizosphere not only inside the liquid or transparent mediums but also in soil or sand [20]. Poorter et al. [42] followed the real-time development of the more complicated horizontal distributions of the root of *Hordeum vulgare.* The morphological and physiological components observed by the MRI might explicate the detected growth patterns and propose an applicable environment for plant development. Previous research obtained the external and internal structures of radishes with distinct visibility of vessels in the xylem and phloem of *Raphanus sativus* by MRI [43]. Afterwards, the MRI was utilized as a technique to clarify the quality of structures and functional properties during subsequent developmental stages [44]. The type of roots of *D. huoshanense* are aerial roots, which are exposed to the moist air to absorb water and nutrients and then transport them through the xylem and phloem to various parts of the plant. There is a hydrophobic barrier between the aerial parts of higher plants and their environment, called the cuticle [45], which contains both epicuticular waxes and intracuticular waxes. The epicuticular waxes will show different morphological types of crystals, such as massive crusts, plates, granules, and tubules with a hollow center [46]. These various structures not only prevent water loss and exogenous attack in plants, but also play a relevant role in building epidermis structures [47]. When attempting to observe the structure of the root of *D. huoshanense* by MRI, we discovered tiny air bubbles on the epidermis (Fig. 2A). We speculated that these could be the type of epicuticular waxes of *D. huoshanense* for growth. What counts is the finding of the detailed structure inside the root, which was uncovered simultaneously when the whole body of *D. huoshanense* was detected by MRI.

In addition, the internal structures of medicinal parts (roots, fruits, seeds, stems, and leaves) need to be evaluated when exploited as raw materials for TCM prescription. Whether their growth conditions are healthy or not exerts a strong influence on the clinical functions of CMMs. The same challenge exists in the medicinal organs of fruits. Detecting the internal structure and quality of fruits in time is a crucial strategy to enhance their functions [48]. Attempts have been made to develop a noninvasive method of evaluation for the internal quality of agricultural products, which can also be exploited as Chinese medicinal materials. Moreover, application modes in agricultural products can provide a new idea for detecting of the quality of CMMs. MRI is one of the most potent strategies to evaluate the internal quality of CMMs based on the accumulated data. Microstructure determines the mechanical and transport properties of fruit tissues and the internal quality of horticultural products. The fruit of the herbal medicine of *Malus pumila* was widely known for its antioxidant, anti-inflammatory, and anti-cancer activities [49]. The algorithm of MRI can efficiently help identify apples with better quality since discriminating between bruised and non-bruised apples utilizes only two scans of the image and simple computations [50]. In 2015, Mazhar et al. [51] made good use of ^1^H-MRI to monitor bruise expression and internal quality over time in *Persea americana* fruit. ^1^H-MRI has also been applied in the determination of the degree of maturity in, for instance, jujube [52]. Data provided from MRI reflected the water status and migration dynamic process of jujube during the blackening process, which improved the internal quality of blackened jujube and had practical significance for promoting the deep processing and industrial development. When there are changes inside the fruit, MRI can also be used to immediately detect quality in the living organism, including *Chaenomeles sinensis* [53]. An answer to the vital issue of detecting the internal conditions of fruits noninvasively was given. Under the premise of ensuring the integrity of the fruit, the measurements obtained by MRI on the dynamics of nutrients inside the organ during storage can provide scientific guidance on quality improvement. According to the studies above, it may be possible to use MRI to directly visualize whether the internal structure and conditions of medicinal parts of CMMs are healthy or not. Moreover, the MRI view of a very dry capsule of *D. huoshanense* (Fig. 2B) is presented here to support the possibility of the above hypothesis. Images uncovered free liquid water content and distribution within the capsule as bright areas; as the higher the free liquid water content, the brighter it appeared. The content of water in the marginal area was higher than that in the center area inside the fruit of *D. huoshanense*. In addition, this may be caused by the presence of more seeds with high lipid content in the center region. This provided intuitive feedback for us to observe the distribution of seeds and the migration of water. Non-destructive imaging from MRI can help us to monitor the ever-changing states in real time during the growing process inside the capsule as it grows, further contributing to better quality control of medicinal organs.

The corresponding author Professor Kai Zhong has applied MRI combined with MRS [54] to examine the internal microstructures of plant organs to reflect their quality more than two decades ago. This provides the feasibility of detecting real-time internal quality conditions of some CMMs whose roots are as medicinal parts. This provides the feasibility of real-time internal quality conditions for detecting of some CMMs whose stems are as medicinal parts. There have also been numerous publications focusing on the applications of MRI in investigating the morphological structure of other tissues and tracking activities, including growth and ripening. The structures of whole pea (*Pisum sativum*) seeds were digitalized for visualization of seed anatomy, which made it feasible to measure the volume and proportional sizes altered over time [55]. The developing processes of barley grains from anthesis to maturity [56] and the formation of distinctive phenotypes of rice seeds exposed to specific conditions were able to be captured by MRI [57]. The tissue architectures of stems and leaves were also ideal for the collection of high-resolution pictures because they suit the MRI probe well. In summary, MRI can image the morphology of the whole plant of CMMs while also obtaining the internal structure and information of individual organs or tissues non-destructively. As traditional medicinal parts, quality control of organs, such as roots and storage fruits, can assure the therapeutic effects of CMMs. MRI bridges the gap by ensuring that data will be accessed by traditional means after the sample has been destroyed. So, there are fewer samples required and more efficient analysis conducted by MRI when measuring the quality of CMMs.

### 3.2 Distribution of active metabolites

CMMs’ quality guarantees their clinical efficacy and is closely related to the pharmacodynamic material basis, i.e., active metabolites. Based on the characteristics of multiple components, CMM can contribute greatly to the medical field by transforming the condition from an abnormal to a normal state [58]. The content and distribution of secondary metabolites differ in CMMs due to environmental and genetic factors, while they are variedly distributed in different medicinal parts of the same CMM [59]. This will have a major impact on their quality and directly affect their efficacy. However, the traditional quality evaluation and component detection method of CMMs generally requires complex pretreatment of the sample (e.g., extraction, separation, and enrichment), and then followed by the use of the LC, GC, LC-MS or GC-MS methods to analyze the chemical composition [60]. The MRI relaxation time curves reflecting the contents of water, starch, lipid, and other components in samples can be obtained in a short time, which is superior to other techniques [5]. It is possible to observe not only the dominant ^1^H resonance of water but also the considerable resonance lines emerging from protons in other active compounds. Resonance lines from protons in chemical compounds are usually differentiated by distinctive chemical shift imaging (CSI). What leads to the various chemical shifts is the distinct shielding effect of the surrounding electrons and nuclei against the external magnetic field. The spatial distribution, transport, and conversion of metabolites in a plant can be mapped well by a series of high-resolution spectra obtained by MRI [28].

#### 3.2.1 Distribution of metabolites in different organs and tissues

The spatial biosynthesis, transport, and metabolism of active metabolites are subject to specific regulation of complex metabolic networks [61]. Phytochemicals in specific regions further lead to various pharmaceutical functions of different botanical parts of medicines. More importantly, there are similarities in the major metabolites between different medicinal parts, suggesting that they can be replaced by each other in some circumstances [62]. To provide references for the development and utilization of CMM when it is considered as a whole, a visual strategy of comprehensively mapping metabolites in medicinal parts and other underutilized tissues will be of high value. Structure images of plants were usually acquired at the end of the CSI, aiming at topographically associating the metabolite spectroscopic data with the matching tissue structure by MRI. Higher concentrations of sucrose, or amino acids in plants, have resonance lines that are especially favorable for spectroscopic MRI measurements [63,64]. It can be dated back to 1994 when MRI was established as a method for noninvasive measurement and localization of sucrose distribution in the Ricinus seedlings [63]. There was a combination of the longitudinal section from the 3D MRI model and metabolite distribution within the embryo sac of growing pea seeds [55]. The content and distribution of sucrose were subtly distinguished from glutamine and alanine when the legume embryo went through the storage process and environmental signals. In addition to sugar and amino acids, the distribution and flow of metabolites such as lipids in plants was another target of the application of MRI. Various lipid maps and related 3D models of seeds and fruits have already been elaborated [65]. Several studies have explored the gradients of lipid storage in developing and living soybean seeds and correlated them with photosynthesis and plastid differentiation in soybeans [66]. Quantitative profiling of the grain *in vivo* demonstrated lipid deposition was mainly comparted in the embryo and the aleurone layer [67]. The same analyses were also conducted for in quantitative imaging of lipids in oilseed rape and fennel mericarps [68,69].

The tissue-specific images of a particular metabolite in the plant can be mapped by MRI, accompanied by rational spatial resolution and the time required for the acquisition of the data. It was also possible to screen various bioactive compounds that were hard to distinguish using the hyphenated analytical methodology of MRI with other techniques for metabolite quantification [70]. Assimilation of nitrate and ammonium in the form of the accumulation pattern of free NH_4_^+^ in *Picea abies* was revealed by ^14^N or ^15^N-MRI. The extent to which the nitrogen source influenced the composition of the free amino acid pool in roots, stems, and needles was examined [71]. A combination of metabolite spectroscopy and morphology imaging of whole plants can provide a chance to holistically visualize both their active compound accumulation and functional tissue information. In the results of MRI experiments conducted on the whole body of *D. huoshanense*, we monitored metabolite profiles of the root and stem. The MRS were processed in jMRUI, followed by raw data were read in MATLAB, and water signal peaks were calibrated (Fig. S1). The reads of all peaks were normalized to get the relative intensities and spectra displayed in Figs. 1B and C. Besides, *D. huoshanense* tested by MRI was also analyzed by a 600 Hz NMR instrument to validate the acquired data referring to the previous study [72]. In detail, the freeze-dried powder of the whole plant of *D. huoshanense* and biological replicates dissolved in D_2_O were detected with ^1^H NMR spectroscopy (Fig. S2). The ^1^H NMR spectra were processed, auto-phased, chemical shift referenced, and baseline calibrated using the Topspin data processing software. The Natural Products Magnetic Resonance Database (NP-MRD) and the Human Metabolome Database (HMDB) were used to search for metabolites in *D. huoshanense*. The characteristic peaks in the ^1^H NMR spectra were assigned to these constituents based on the database and the reported chemical shifts for the metabolites in *D. huoshanense*, which have been added in Table S1. Then the NMR database of the constituents in *D. huoshanense* was built up, and the total correlation spectroscopy (TOCSY) ^1^H–^1^H spectrum and heteronuclear single-quantum coherence (HSQC) ^1^H–^13^C 2D spectra were acquired for validation (Fig. S3). Since the MRS data of the root and stem were obtained under the same conditions and in the same plant, the normalized integral value of these characteristic peaks represented the relative content of the chemical components [73]. These compounds with different contents and related pharmacological effects were also annotated aligning with peaks acquired by ^1^H NMR metabolomics (Table 2) [[74], [75], [76], [77], [78], [79], [80], [81], [82], [83], [84], [85], [86], [87], [88], [89], [90], [91], [92], [93], [94], [95], [96], [97], [98], [99], [100], [101], [102], [103], [104], [105]]. All structures of compounds in Table 2 were drawn as shown in Fig. 2C. A greater variety of chemical constituents were obtained in the stem than in the root. Furthermore, the integral value of most characteristic peaks was also significantly higher in the stem than in the root. It may be the reason why the stem *D. huoshanense* is usually the main medicinal parts when applied in clinical treatments.

**Table 2:** The chemical constituents with different contents in the stem and root of *Dendrobium huoshanense* (*D. huoshanense*) detected by magnetic resonance imaging (MRI).

Number	Chemical shifts (ppm)	Compounds	Pharmacological effects	Refs.
1	0.98	(3*R*,6*R*)-3-Hydroxyl-α-ionone	Anti-inflammatory activity	[74,75]
2	1.00	(*S*)-4-Isobutyl-3-oxo-3,4-dihydro-1H-pyrrolo[2,1-c][1,4]oxazine-6-carbaldehyde	Antivirus activity	[76,77]
3	1.40	Grasshopper ketone	Anti-inflammatory, antitumor, and neuroprotective activities	[78,79]
4	2.59	Dihydroconiferyl dihydro-*p*-coumarate	Acetylcholinesterase inhibitory and antioxidant activities	[80,81]
5	2.63	Dendronbibisline B	Anti-tumor activity	[82]
6	2.68	Naringenin	Antivirus, anti-inflammatory, anti-cancer, and neuroprotective activities	[83,84]
7	2.70	Dihydroresveratrol	Anti-inflammatory, anti-cancer, and intestinal protective activities	[85,86]
8	2.75	Batatasin III	Anti-inflammatory and antitumor activities	[87,88]
9	2.80	Coniferyl *p*-coumarate	Antioxidant activity	[89,90]
10	3.18	(+)-Syringaresinol	Neuroprotective and anti-inflammatory, antioxidative, and α-glucosidase inhibitory activities	[91,92]
11	3.44	Lirioresinol A	Antivirus and anti-inflammatory activities	[93,94]
12	3.45	*N*-*trans*-Coumaroyltyramine	Anti-inflammatory, acetylcholinesterase inhibitory, and antimycobacterial activities	[95]
13	3.47	*trans*-*N*-Feruloyltyramine	Anti-inflammatory, anti-tumor, and antioxidant activities	[96,97]
14	3.50	(+)-Lyoniresinol	Tyrosinase inhibitory, neuroprotective, and anti-cancer activities	[98,99]
15	3.62	*p*-Methoxycinnamic acid	Anti-inflammatory, anticancer, anti-atherosclerotic, and neuroprotective activities	[100,101]
16	3.82	4,4′-Dihydroxy-3,5-dimethoxybibenzyl	Anti-cancer activity	[102]
17	4.17	Medioresinol	Anti-inflammatory and prevent ischemic stroke activities	[103,104]
18	4.58	Sinapyl *p*-coumarate	No pharmacological reports	[105]

#### 3.2.2 Distribution of metabolites in similar species of CMMs

Not only the different parts of one CMM but also the differences between it and its similar species can be visualized by MRI. The sites of lipid deposition and images of the anatomy of layers parallelly analyzed offered the possibility of linking lipid accumulation to seed development. This approach has been used in two similar oat cultivars, with different colors used to represent different concentrations of lipids [106]. Differences of them in the principal location of lipid deposition and content were discovered, and it further highlighted the difference in the relationship between energy storage and carbon allocation. This approach can also be applied to distinguishing very similar species of CMMs and seeking quality markers. Similar species of CMMs are easily confused and sometimes cannot be distinguished clearly on the basis of morphology. If the distribution of the active constituents can be combined, it is conducive to CMM identification. The differences between *D. huoshanense* and its similar species *Dendrobium moniliforme* *(D. moniliforme)* are hard to distinguish due to their similar morphology. The MRI performed on these two species and their biological replicates can be applied to clarify their differences in active metabolites. The main metabolites distributed in the roots of these two species were found to be various. There were significant differences in the contents of chemical components at 1.00 and 3.50 ppm (Fig. 2D). ^1^H NMR detections of *D. huoshanense* and *D. moniliforme* were also obtained to verify the data from the MRI. Displayed in Fig. S4, it can be found that the relative intensities of the two compounds with chemical shifts at 3.5 and 1.0 ppm in these two species were consistent in the MRI and NMR measurements. And we made it possible to detect comprehensive changes between different parts of one variety or between similar varieties by measuring multiple indicators noninvasively. This can help us to determine the material basis of CMMs in conjunction with related medicinal parts mentioned above to obtain more comprehensive data. As an imaging technology, MRI can not only visualize the overall microstructure of CMMs but also obtain the NMR data of metabolites to understand the differences in the types and contents of compounds between different tissues or plants. Therefore, MRI has the potential to evaluate and inspect the quality of CMMs through direct qualitative and quantitative analyses of the marker components of medicinal materials and, at the same time, enable the identification of potential active substances.

### 3.3 Visualization of dynamic changes in CMMs

The quality of CMM is closely related to its phenological period, special processing, and environment. The accumulation and mutual transformation of active components show a dynamic trend with the growth and processing of CMM. As reviewed above, MRI enables the non-destructive imaging of the whole body and tissue and the real-time spatial distribution of compounds to colocalize them with their botanical structures. The prominent effects of CMMs are based on their optimal character and high quality, which require real-time detection during dynamic changes by MRI.

#### 3.3.1 Visualization of dynamic changes throughout the growth and development

The quality of CMMs is influenced by various conditions, such as the harvesting period and the year of growth. The contents of soluble saccharides and amino acids kept on changing significantly with the age of *Coptis chinensis*, as reported [107]. The harvest period (including the harvest year, month, or even day) of medicinal parts is one of the critical factors affecting the quality of TCM [108]. It has certain rules to follow, namely, the overall evaluation between the dynamics of the accumulation of active components and the yield of medicinal parts. However, these two indicators are sometimes inconsistent, which makes it urgent for real-time detection of dynamic changes of them both in CMM. MRI enables visualization of metabolite distribution throughout plant growth. The developing processes of barley grains from flowering to maturity were visualized in terms of changes in lipid and other metabolite distribution in organs and tissues [56]. Verscht et al. [109] displayed the accumulation pattern of sucrose in the phloem of Ricinus seedlings and changes between normal and starvation conditions (sucrose deprivation or a cotyledon petiole break-off). The data offered by MRI enabled the direct connection between structural and metabolite imaging of dynamic chemical compounds to precisely identify and localize metabolites within the tissues of plants during the whole growth and development process [110]. This can be used to determine the optimal harvesting period and medicinal parts.

#### 3.3.2 Visualization of dynamic changes during special processing

**Fig. 3:**
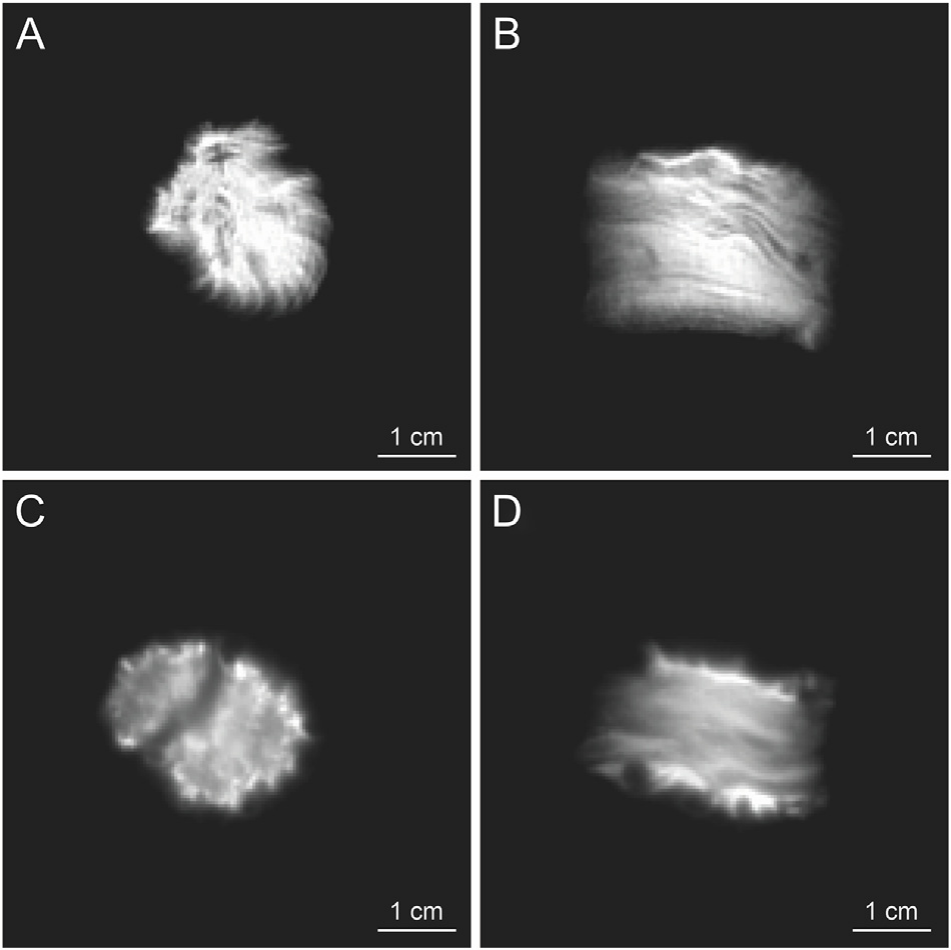
Differences of *Salvia miltiorrhiza* during sweating processing revealed by magnetic resonance imaging (MRI). (A, B) Axial (A) and sagittal (B) images of the root before sweating processing. (C, D) Axial (C) and sagittal (D) images of the root after sweating processing.

The medicinal plant needs to be concocted and processed before being applied to TCM preparation, during which active compounds dynamically change. Traditionally, raw and steamed *Panax notoginseng* possess respective pharmacological functions, while there are spatiotemporal changes of metabolites in a particular part during the steaming process [111]. Accurately and noninvasively visualizing the spatiotemporal variation of metabolites during CMM processing is of great significance for clarifying the pharmacological effects of medicines. The moistening process of *Rehmanniae Radix* was detected quantitatively by low-field nuclear magnetic resonance and imaging (LF-NMR/MRI) technology [112]. It attempted to elucidate the scientific indications of the moistening process by investigating changes in water absorption and expansion kinetics. In our review, the morphological and material basis changes of the roots of *Salvia miltiorrhiza* during sweating processing were also revealed by MRI (Fig. 3). It further shed light on the dynamic metabolite transport and exchange of medicinal plants *in situ* during some unique processes through straightforward co-registration of MRI technique and isotope-labeling trace. Structural imaging and metabolite analysis before and after concoction can help clarify the special quality formation of CMMs.

The quality of CMMs can be largely influenced by the environment. Imaging techniques may help visualize plant changes under environmental factors, whether they are caused by the accumulation of active constituents and their effects on functions or structural alterations [113]. MRI can also be applied in clarifying the quality formation of plants in specific environments [114]. It can holistically elucidate the real-time response to specific stresses of plants. Instead, conventional physiological trials are inclined to emphasize the response generally applied to specific organ or tissue. The other problem is that only one stress is generally used to simulate the environment of CMM, which may be the interaction of a multitude of stresses during growth. MRI of plant physiology *in situ* may well be the solution to this problem [115]. MRI can do a favor in claiming water transport and dynamics when various species are exposed to drought circumstances. It allowed visualization of changes taking place in plants *Quercus ilex* that experienced varying degrees of drought [116]. MRI can be employed to uncover processes and mechanisms in CMMs that are already highly adapted to drought environments. Inspired by these studies, MRI can also be exploited to be a better alternative device for the identification of individuals with superiority about water use efficiency and quality. Besides, the detailed responses to subzero temperatures of plants and their single organs were carried out by MRI as well [117]. Their tolerance to cold was primarily reflected in the fact that the water or sucrose was transported slowly. Furthermore, procedures have also been developed based on MRI to observe how various tree species deal with cold stress *in situ* [118]. This implies a potential application of MRI in exploring how the special quality of CMM forms under a specific environment.

## 4 Challenges and perspectives

Although MRI shows promise for the future task of evaluating the quality of CMMs, there are several technical challenges encountered. On one hand, the expensive device has hindered the prevalence of MRI. Low-cost MRI have been developed in experiments, but their resolution is far lower than that of other devices. On the other hand, in general circumstances, the imaging time significantly increases as the resolution requirement rises. To overcome these challenges, it is crucial to primarily focus on developing high-resolution and effective MRI devices based on preliminary theoretical research. Specific coils, probes, and equipment can be created or improved for a particular CMM plant to achieve functional visualization with higher resolution and less time. CMMs of high quality are often accompanied by the optimal morphology. Using more specific devices and coils for a particular CMM can make it possible to better identify its unique morphological characteristics.

Additionally, MRI can also be applied to more studies of CMM in accordance with its ability to image CMM structures and analyze dynamic distributions of metabolites. For example, 1) revealing the dynamic transport of the specific compound in a single plant during the whole growth and development process. The MRI technique, combined with stable isotope labeling, can track specific metabolite molecules uptake and exchange in plants in real-time, *in situ*, and non-destructively [119]. ^13^C and ^23^Na can be applied to acquire NMR metabolic information and imaging associated with specific molecules or their metabolic derivatives [120]. As multiconstituents are rich in CMMs, stable labeling associated with MRI may offer a new perspective for proteins and polysaccharides with known structures. Thus, MRI may make some achievements in announcing the morphological and compositional changes of typical metabolites in a single living CMM during the growth and development stage based on its non-destructiveness. 2) CMMs in clinical use is not just a form of direct use. Many CMMs are processed into various forms of pharmaceutical preparations to facilitate taking or to increase efficacy and reduce toxicity. The quality inspection of such Chinese patent medicines prepared into capsules and tablets is also an essential part of the safety and effectiveness of the clinical medication. The current monitoring methods need to destroy these samples and then use various chromatographic or MS techniques for evaluation, which are cumbersome, time-consuming, and laborious. According to the characteristics of MRI, it may be possible to use MRI to directly visualize the internal composition information of these drugs to achieve the purpose of rapid detection of drug quality. 3) Establishment of a visual database on the 3D structures and chemical compositions of CMMs in different environments and ages has epoch-making significance in the fields of Chinese medicine identification, Chinese medicine chemistry, and Chinese medicine quality evaluation and inspection. MRI technology exhibits great potential in the quality evaluation of CMMs, specifically focusing on visualizing the structures and distribution of metabolites during dynamic processes. Nonetheless, less research has been conducted on the dynamics of metabolites absorption, distribution, metabolism, and excretion (ADME) of CMMs in animals by MRI. Combining the applications in animals by MRI with the comprehensive database can contribute to elucidating the quality and efficacy of CMMs. 4) Multimodal imaging combinations can help visualize the quality of CMMs more comprehensively. Optical imaging is a real-world technique for directly observing the microstructure of matter but is limited by penetration depth. It is possible to couple MRI with optical imaging to integrate their advantages, such as a holistic view of visualization and intuitive characteristics, which may make an unexpected breakthrough in the field of imaging. Moreover, imaging techniques with high spatial resolution (e.g., MRI and computed tomography (CT)) were often integrated with others with high sensitivity (e.g., PET and fluorescence imaging) to provide more detailed information about several diseases. The proper selection of multimodal imaging combinations will grant more powerful approaches to the visualization for the quality of CMMs and the more innovative application of MRI.

## 5 Conclusions

The review discusses the potential of MRI technology for studying CMMs. This study highlights the capability of MRI to model the internal and external structures of CMMs, visualize the compartmentalization and transport of metabolites, track the dynamics of metabolism, and detect the quality of CMMs in specific environments. Overall, MRI entirely opens up a new perspective for non-destructively accessing CMMs’ quality *in situ* over time. With discrepancies exist in shapes and sizes, different CMMs only need to be replaced with the corresponding coils and measuring parameters when they are being observed. The set of MRI devices can be commonly used in different fields, leading to fewer costs and wider applications. As a newly introduced technology, the non-destructive nature of MRI enables repeated imaging experiments throughout the lifetime of a CMM, allowing for comprehensive monitoring of their morphology, active constituents, and response to various processes. Multiple pieces of information would be controlled *in situ* simultaneously using the method, laying the cornerstone for comprehensive analysis and precise quality evaluation. This appears to be an efficient and accurate method urgently needed in the modernization and development of TCM. Moreover, the factors mentioned above are directly responsible for the unique quality of the CMM. The key to holistic quality visualization of traditional CMMs lies in accurately measuring and systematically combining their typical traits of them, which can be accomplished by MRI as reviewed here. Potential application modes of MRI for visualizing the quality formation and evaluation of CMMs have been put forward. Based on existing research, MRI is expected to find expanded use in the future, suggesting that the development of a complete MRI-detecting methodology would require collaboration among multiple disciplines and organizations. By integrating a cooperative strategy, the adoption and utilization of MRI technology can be accelerated, further enhancing its role in visualizing the quality of CMMs.

## CRediT authorship contribution statement

**Jing Wu:** Validation, Writing – original draft. **Kai Zhong:** Conceptualization, Writing – review & editing. **Hongyi Yang:** Methodology. **Peiliang Zhang:** Data curation. **Nianjun Yu:** Resources. **Weidong Chen:** Resources. **Na Zhang:** Data curation. **Shuangying Gui:** Supervision. **Lan Han:** Supervision. **Daiyin Peng:** Conceptualization, Funding acquisition, Project administration.

## Declaration of competing interest

The authors declare that there are no conflicts of interest.
