# Supplementary material for: A holistic visualization for quality of Chinese materia medica: Structural and metabolic visualization by magnetic resonance imaging
Source: J Pharm Anal. 2024 Jun 8;14(11):101019. doi: 10.1016/j.jpha.2024.101019 (PMC11696849; doi:10.1016/j.jpha.2024.101019)
Supplement: Multimedia component 1 [file mmc1.docx]

Supplementary Materials

**Table S1** Chemical shifts and splitting patterns of the peaks used in peak assignments.

| Number | Chemical shift (ppm) | Splitting type | Metabolite |
| --- | --- | --- | --- |
| 1 | 0.62 | m | Citric acid |
| 2 | 0.98 | s | (3*R*,6*R*)-3-Hydroxyl-α-ionone |
| 3 | 1.00 | s | (*S*)-4-Isobutyl-3-oxo-3,4-dihydro-1H-pyrrolo[2,1-c][1,4]oxazine-6-carbaldehyde |
| 4 | 1.12 | d | Rutin |
| 5 | 1.40 | m | Grasshopper ketone |
| 6 | 1.77 | m | Dihydroconiferylalcohol |
| 7 | 1.96 | t | Arachidonic acid |
| 8 | 2.14 | m | Methyl pyroglutamate |
| 9 | 2.18 | m | Tetrahydro-5-oxo-2-furancarboxylic acid methyl ester |
| 10 | 2.26 | t | Methyl-3-propionate |
| 11 | 2.36 | t | Glutaric acid |
| 12 | 2.59 | s | Dihydroconiferyl dihydro-*p*-coumarate |
| 13 | 2.63 | s | Dendronbibisline B |
| 14 | 2.68 | dd | Naringenin |
| 15 | 2.70 | s | Dihydroresveratrol |
| 16 | 2.75 | dd | Batatasin Ⅲ (8)^[91]^ |
| 17 | 2.80 | m | Coniferyl *p*-coumarate |
| 18 | 3.18 | d | Syringaresinol |
| 19 | 3.23 | d | L-Arginine |
| 20 | 3.44 | d | Lirioresinol A |
| 21 | 3.45 | d | *N*-trans-Coumaroyltyramine |
| 22 | 3.47 | t | Trans-*N*-Feruloyltyramine |
| 23 | 3.50 | dd | Lyoniresinol |
| 24 | 3.62 | m | *p*-methoxycinnamic acid |
| 25 | 3.71 | m | 4-O-β-D-glucopyranosyl coniferyl aldehyde |
| 26 | 3.82 | m | 4,4’-Dihydroxy-3,5-dimethoxybibenzyl |
| 27 | 3.89 | dd | Gastrodin |
| 28 | 4.17 | d | Medioresinol |
| 29 | 4.58 | m | Sinapyl *p*-coumarate |


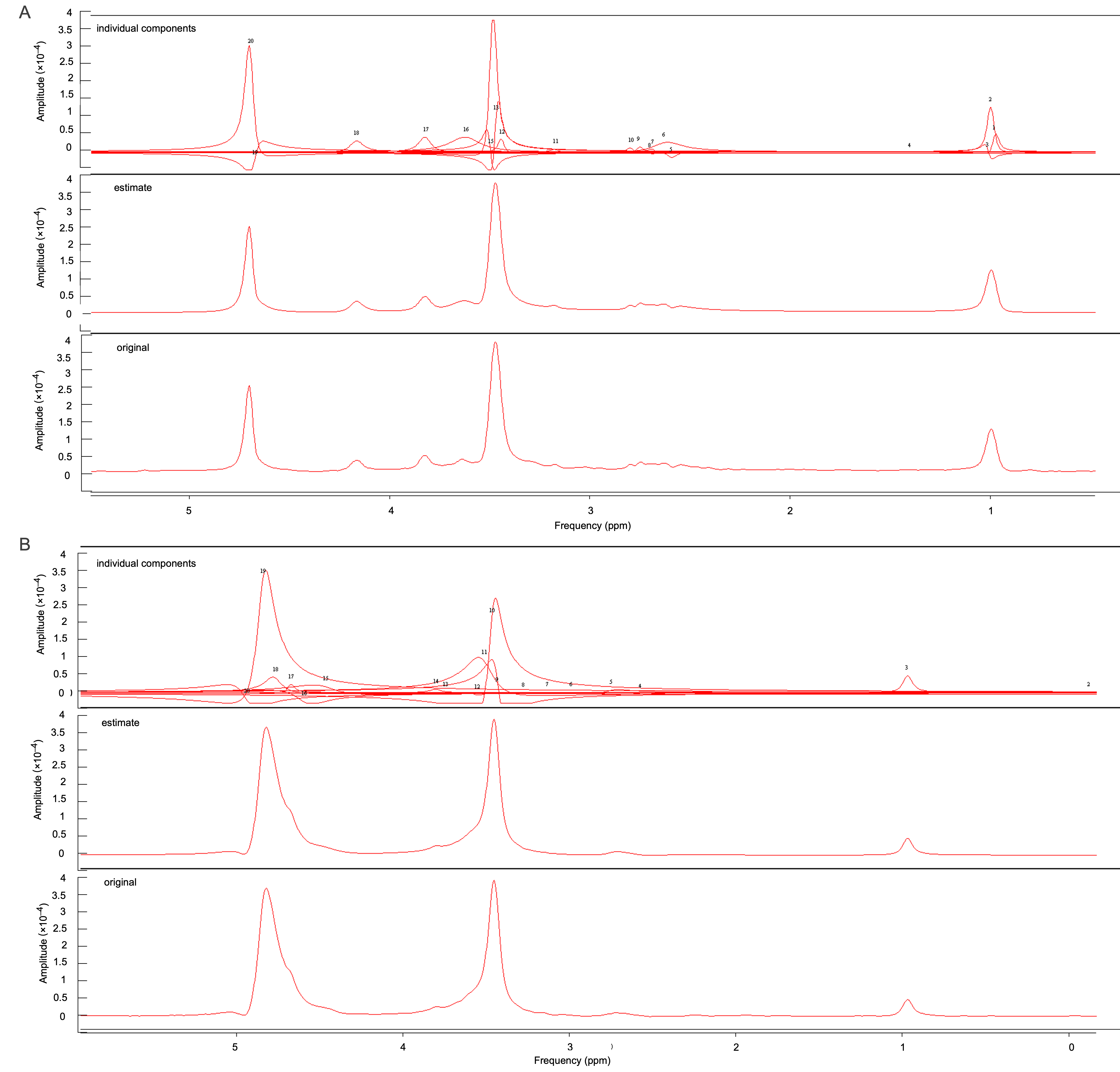


Figure S1. Magnetic resonance spectroscopy of the root (A) and stem (B) of *Dendrobium huoshanense.*


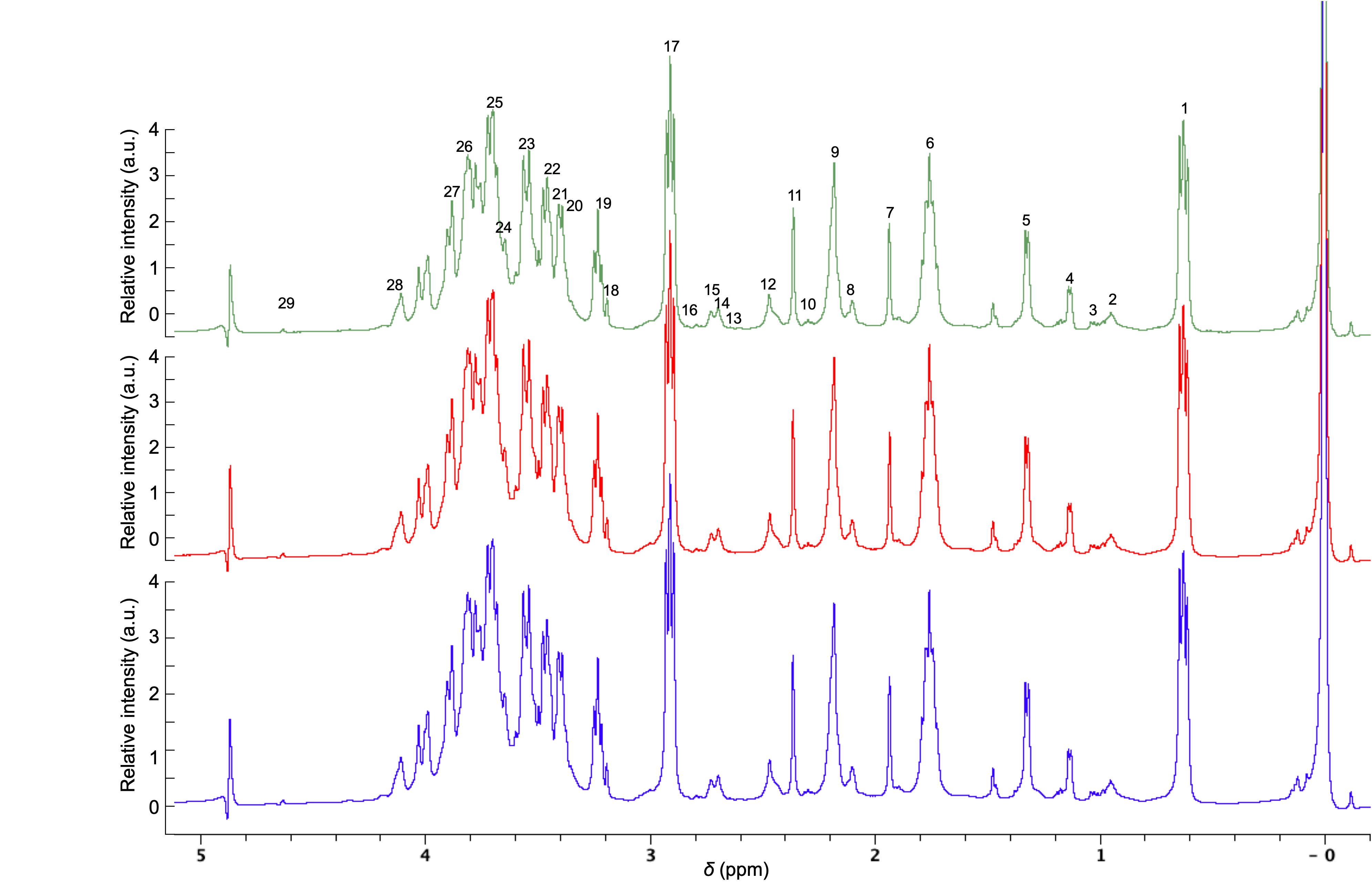


Figure S2. ^1^H nuclear magnetic resonance spectrums of freeze-dried powders *Dendrobium huoshanense* with peaks assignments.


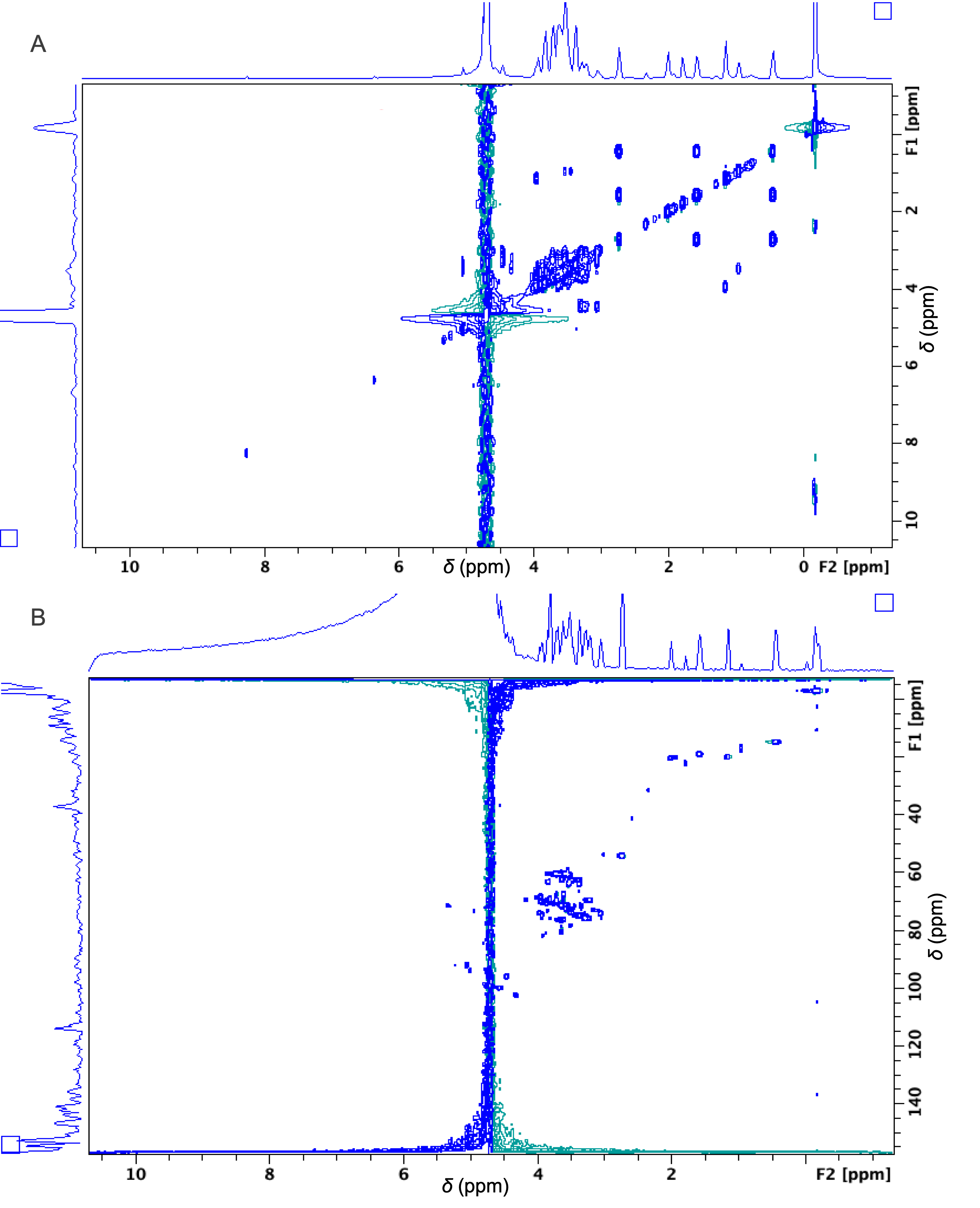


Figure S3. ^1^H−^1^H TOCSY spectral profile (A) and ^1^H−^13^C HSQC (B) spectral profile of *Dendrobium huoshanense.* TOCSY: total correlation spectroscopy, HSQC: heteronuclear single-quantum coherence.


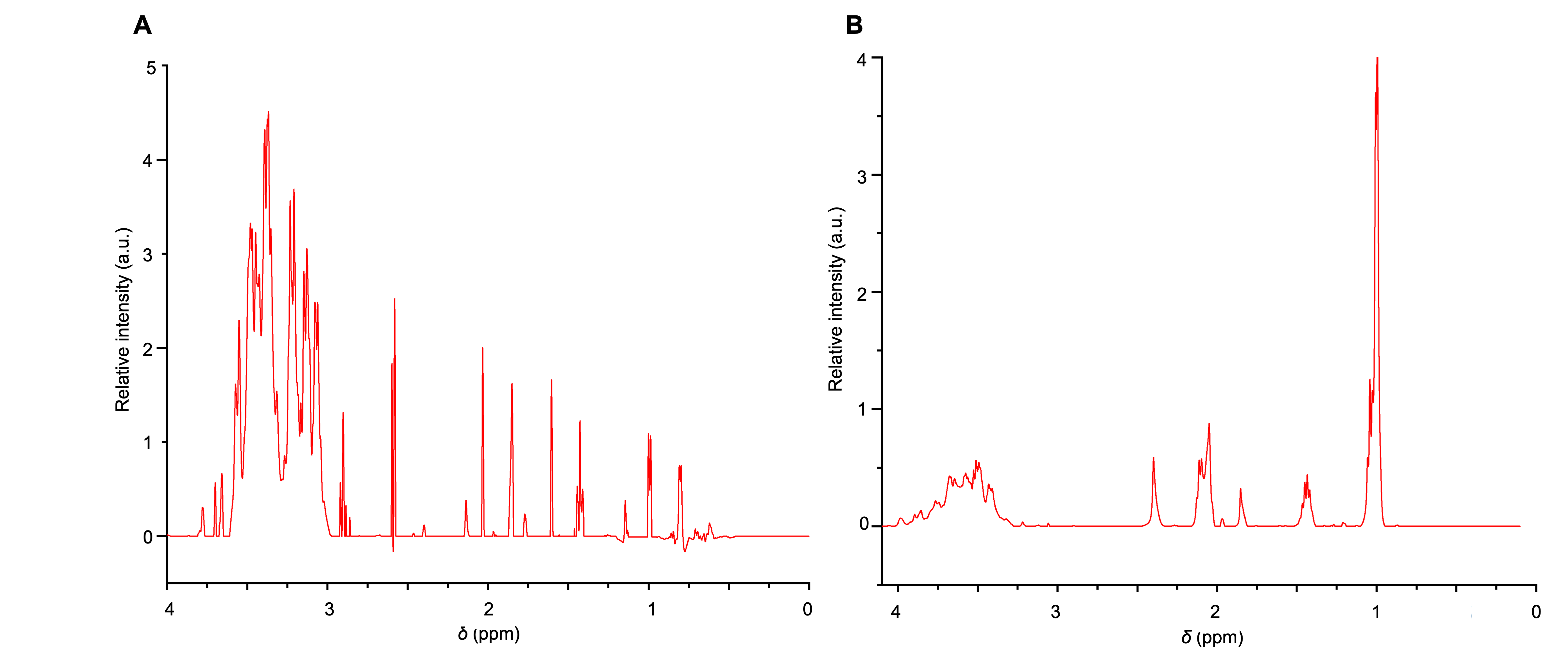


Figure S4. ^1^H nuclear magnetic resonance of freeze-dried powders of *Dendrobium huoshanense* (A) and *Dendrobium moniliforme* (B).
